# Beliefs about weight and breast cancer: an interview study with high risk women following a 12 month weight loss intervention

**DOI:** 10.1186/s13053-014-0023-9

**Published:** 2015-01-09

**Authors:** Claire E Wright, Michelle Harvie, Anthony Howell, D Gareth Evans, Nick Hulbert-Williams, Louise S Donnelly

**Affiliations:** Department of Clinical Sciences and Nutrition, University of Chester, Chester, CH1 4BJ UK; Nightingale & Genesis Breast Cancer Prevention Centre, University Hospital South Manchester NHS Foundation Trust, Southmoor Road, Manchester, M23 9LT UK; Genomic Medicine, Manchester Academic Health Sciences Centre, University of Manchester & Central Manchester Foundation Trust, Manchester, M13 9WL UK; Department of Psychology, University of Chester, Chester, CH1 4BJ UK

**Keywords:** Breast cancer, Risk reduction behaviour, Qualitative, Oncology, Hereditary, Body weight, Interview

## Abstract

**Background:**

Breast cancer is the most common cancer in the UK. Lifestyle factors including excess weight contribute to risk of developing the disease. Whilst the exact links between weight and breast cancer are still emerging, it is imperative to explore how women understand these links and if these beliefs impact on successful behaviour change.

**Method:**

Overweight/obese premenopausal women (aged 35–45) with a family history of breast cancer (lifetime risk 17–40%) were invited to a semi-structured interview following their participation in a 12 month weight loss intervention aimed at reducing their risk of breast cancer. Interviews were carried out with 9 women who successfully achieved ≥5% weight loss and 11 who were unsuccessful. Data were transcribed verbatim and analysed using thematic analysis.

**Results:**

Three themes were developed from the analysis. The first theme *how women construct and understand links between weight and breast cancer risk* is composed of two subthemes, *the construction of weight and breast cancer* risk and *making sense of weight and breast cancer risk*. This theme explores women’s understanding of what contributes to breast cancer risk and whether they believe that weight loss could reduce their breast cancer risk. The second theme *motivation and adherence to weight loss interventions* explains that breast cancer risk can be a motivating factor for adherence to a weight loss intervention. The final theme, *acceptance of personal responsibility for health* is composed of two subthemes *responsibility for one’s own health* and *responsibility for family health* through making sensible lifestyle choices.

**Conclusion:**

Beliefs about weight and breast cancer risk were informed by social networks, media reports and personal experiences of significant others diagnosed with breast cancer. Our study has highlighted common doubts, anxieties and questions and the importance of providing a credible rationale for weight control and weight loss which addresses individual concerns. Counselling and health education material should be tailored to facilitate understanding of both genetic and modifiable risk factors and should do more help individuals to visualise the weight and breast cancer link.

## Introduction

Breast cancer is the most common cancer in the UK with 49,564 women newly diagnosed in 2010 [1]. A combination of reproductive factors, family history, and lifestyle factors including excess weight has a significant impact on the risk of developing the disease [2]. The exact links between weight and breast cancer are complex and still emerging [3,4]. Expert reports recommend the prevention of weight gain and maintenance of a healthy weight to reduce the risk of breast cancer and other diseases [2,5,6]. Observational studies indicate that gains of 5-12 kg during pre- and postmenopausal years increases risk of postmenopausal breast cancer by 50% or more compared with those who maintain a healthy weight [7-9] and that modest weight loss in pre- and postmenopausal years (5-10%) can reduce breast cancer risk by 25-40% [8,10]. Intervention trials report reduced levels of breast cancer related hormones with modest weight loss (5-10%) in both pre- and postmenopausal women, including reductions in oestradiol, inflammatory markers, and change in adipokines [11].

Effective weight control interventions may help attenuate the rising breast cancer incidence within the general population (10-12% lifetime risk) [2,5] women with a family history of breast cancer and moderate/high risk (17-40% lifetime risk) [12-14] and women with high risk gene mutations (such as *BRCA1/2*) (60-80% lifetime risk) [15,16]. General population surveys report low awareness (5-12%) of the links between obesity and lifestyle factors and breast cancer risk [17]. A survey of women in a UK Breast Cancer Family History Clinic (FHC) estimated that 67% were aware that obesity increases breast cancer risk, however, these women are not routinely given advice and support with weight control within this setting [18]. Indeed, women under the care of a FHC may have greater adiposity than average women [19]. Little is known about how women with a family history of breast cancer would respond to advice and support with weight loss as a strategy to reduce their cancer risk. Some studies suggest that an increased genetic breast cancer risk can motivate healthier lifestyle choices [20] but this is not consistent [21] and more research is needed to understand why some women do not respond to this risk reducing advice [22].

This qualitative interview study was undertaken with 20 premenopausal women who have a family history of breast cancer (≥17% lifetime risk) but are not gene mutation carriers. They were recruited after taking part in a 12 month intervention study to examine the effects of weight loss on biomarkers of cancer risk [23,24]. These women had been advised that their postmenopausal breast cancer risk was further increased due to significant adult weight gain (>7 kg since age of 20 years) which could potentially be reduced with weight loss [8,9]. The aims of this interview study were to explore women’s perceptions and experiences of managing breast cancer risk after weight loss advice.

## Method

### Design and participants

Women were recruited following participation in a study on the effects of weight loss on cancer risk biomarkers [23,24]. Participants were overweight or obese, had gained weight in adult life (>7 kg gain), were premenopausal, aged 35–45 years and had a family history of breast cancer (lifetime risk 16–40%, Tyrer-Cuzick [25]) but were not known *BRCA1/2* carriers. They had been asked to follow a 12 month diet and exercise weight loss intervention (25% energy restricted Mediterranean diet and 150 minutes moderate activity/week). Each participant received an individualised diet and a home based exercise plan from the study dietitian and exercise specialist. To maximise compliance, they were asked to attend a weekly group exercise session for the first 12 weeks, and monthly appointments with the study dietitian throughout the 12 month intervention to assess change in weight and reinforce diet and exercise recommendations. A range of cognitive behavioural techniques such as self-monitoring, obtaining peer/family support and stimulus control were encouraged to increase compliance. Participants had been counselled on the links between weight and breast cancer prior to entering the study by doctors in the FHC and this was reinforced on entry to the trial and throughout the trial with monthly one-to-one advice sessions with the specialist breast cancer dietitian. Women in the intervention were advised that their risk of developing breast cancer was greater because they had gained weight in adult life (>7 kg gain) and that this risk could potentially be reduced with modest weight loss (≥5%). Women were deemed successful if they had lost ≥5% of their total body weight during the weight loss intervention. All participants had an exit consultation with the study physician at the end of the weight loss trial with feedback on changes in hormone levels and their potential risk reduction. This feedback was undertaken prior to taking part in the interview study.

Forty women from the weight loss intervention were invited to take part in this interview study. Twenty women agreed to participate (50% response rate), including nine women who had achieved the target weight loss (≥5%) and 11 who had not. A topic guide was developed from existing literature and interviews were conducted to explore women’s beliefs about weight and breast cancer, whether these beliefs motivated weight loss, and the experience of participating in a weight loss intervention for breast cancer risk management. Interviews lasted between 90–180 minutes and were carried out at a location of the participant’s choice. Nineteen interviews were conducted in the participant’s home and one at the FHC. Ethical approval was obtained from the South Manchester Ethics Committee (reference 03/SM/159).

### Typical questions used to guide interview

- Why did you participate in study looking at weight loss and breast cancer risk?
- Tell me about your family’s experience of breast cancer
- Do you think that weight and breast cancer are linked? Why do you think that?
- Did risk counselling change your lifestyle choices?
- How do you feel about weight monitoring since the study?
- Have you discussed this risk factor with your family?

### Analysis

Interviews were transcribed verbatim and data were managed using the QSR NVivo 9 package (QSR international). Data were analysed using thematic analysis [26]; this method is not subject to a pre-existing coding frame but is open-ended, exploratory and driven by the data. Each transcript was read several times and themes salient to the individual’s experience were highlighted. For each transcript we aimed to capture the meaning that each individual’s experience conveyed. Following these initial readings and coding of the entire corpus of data, the lead analyst (CW) met with the members of analytic team (LD, NHW) to discuss emergent themes and areas for deeper exploration. Individual themes were then compared across all transcripts, taking into account convergent and divergent experience. Overall themes that best describe women’s understanding of weight loss for breast cancer risk reduction formulate the analysis. Each theme is supported by the richest data extracts available from the transcripts in order to convey the central message of that theme.

## Results

Participant characteristics are outlined in Table 1. Three themes are presented in the analysis;

**Table 1:** **Subject characteristics (n = 20)**

Estimated lifetime breast cancer risk from Tyrer-Cuzick model (%) [25]	
≥30%	2 (10%)
25-29%	4 (20%)
20-24	7 (35%)
≤19	7 (35%)
Weight gain since 20 years of age	
7-15 kg	10 (50%)
16-25 kg	4 (20%)
≥26	6 (30%)
Social deprivation^†^	
1 - 2	13 (65%)
3 - 4	6 (30%)
5*	1 ( 5%)
Weight change of interviewees during 12 month intervention study	
>5% weight loss	9 (45%)
0-5% weight loss	6 (30%)
Weight gain	3 (15%)
Dropped out	2 (10%)

^†^Based on Index of Multiple Deprivation score quintiles for Greater Manchester at Lower Layer Super Output Areas 2010.

*People in most deprived areas.

1. *How women construct and understand links between weight and breast cancer risk,*
2. *Motivation and adherence to weight loss interventions* and
3. *The acceptance of personal responsibility for health*.

### Theme one: how women construct and understand links between weight and breast cancer risk

#### The construction of weight and breast cancer risk

Most participants constructed their knowledge about weight and breast cancer risk from examples of breast cancer in others they knew personally, or stories reported in the media. In the quote below Elaine cites media reports of lower breast cancer risk in countries with lower average body weight; she reports finding this credible until she attempts to validate this message with her slim mother’s experience of breast cancer.

*I’ve read articles where people in Japan, for example, who as a nation aren’t as fat as we are and have a lower incidence of breast cancer, so I thought well there must be some truth in it (Elaine, 42y, weight stable with the intervention).*

However, when asked by the interviewer how *convinced* she was by this, she went on to add,

*Not totally because Mum didn’t smoke or drink or do anything like that, she did yoga, ate organic food and where did it get her? So I’m managing my life, I’m living my life how I want to and so what? I’d like to see some more proof (Elaine, 42y, weight stable with intervention).*

Thus, where family experience appeared to contradict medical evidence, women held discordant beliefs about the link between weight and disease, leading to a sense of confusion about the exact causes of breast cancer. A diagnosis of breast cancer in the family influenced women’s priorities and lifestyle choices in different ways. Some had a strong sense that life was short and precious and wanted to live their life to the full. Others wished to take all possible action to reduce their risk. Reflecting on a slim relative or friend who had developed cancer led to doubts about the relationship between weight and breast cancer. Women tended to focus on personal observations or information which appeared to reduce the significance of the cancer risks associated with excess weight, as Lorraine describes below***.***

*Well there’s so many people with breast cancer that, that aren’t overweight. I know my mum wasn’t overweight and never had been, I’m not convinced, no. (Lorraine, 44y, 4% weight gain with the intervention).*

#### Making sense of weight and breast cancer risk

Women identified a need for a united and clear message on how and why weight gain increases risk of breast cancer. As Deborah explains, this would be required if they were to fully accept that weight management may have a role in managing their risk.

*I believed completely that for women who had gained weight there was an increased risk of breast cancer but I wasn’t totally convinced and I’m still not that’s it’s the weight that causes the increased risk. I want to know a lot more about it, I want to know what it is about those women that’s made them gain weight and if that that’s causing the increased risk of cancer (Deborah, 44y, weight stable with intervention).*

Participants spoke of exactly how they considered weight and breast cancer to be linked. Cathy speculated that body fat “fuelled” cancer growth and that a fit body or that healthy weight would be better able to “defend” itself from disease.

*Your body [at a healthy weight] would be better able to generate healthy cells and fight invading cells and again if you go back to evolution people must have been pretty fit, you know, they must have been pretty fit (Cathy, 42y, weight stable with intervention).*

The lack of a clearly defined mechanism of how weight causes breast cancer made it difficult for many women to conceptualise the link between weight loss and their breast cancer risk. All held the view that weight loss was difficult. For women, like Amanda, this appeared to be potential disincentive to their weight loss efforts and one of the ways they justified their delay in tackling their weight.

*If you said chocolate, cheese and chips increase my risk of breast cancer then I would never eat them again… but it’s because it isn’t specific I think it’s really easier to ignore it and think oh I’ll do it tomorrow, I’ll do it another day. I need someone to say yes it is definite then I’m fairly sure I would lose some weight* (Amanda, 44y, weight stable with intervention).

### Theme two: motivation and adherence to weight loss interventions

Over the course of the study, participants developed a greater understanding of the research linking weight, hormone levels and breast cancer, through both study information and outside resources helping them to maintain motivation to achieve weight loss target over the year-long intervention.

*If I hadn’t wanted to lose weight for other reasons as well I might not have been 100% convinced that it was worth it at that stage, but the more I've looked into it now having been involved in this study I think it’s quite a big part. It’s not my only motivating factor in what I'm going to eat, but it’s there and that’s good, it keeps reminding us (Laura, 45y, 7% weight loss with intervention).*

Women who successfully lost weight primarily did so to improve confidence and appearance, and also for their general health. Believing that they could exert some control over their breast cancer risk was a secondary motivation for weight reduction. Some of our participants viewed their breast cancer risk as unchangeable because of their family history, yet still valued general disease risk management information including weight loss advice and support.

*It’s quite helpful really, [its] another motivation. Fat people like me need a lot of motivation. I didn’t find it a negative….you cannot remove your body make-up unfortunately but you know I don’t think you should be devoid of all responsibility; it’s up to you to do something about it (Pamela, 46y, 7% weight loss with intervention).*

### Theme three: acceptance of personal responsibility for health

#### Responsibility for one’s own health

For women who achieved their target weight loss, the possible link between weight and breast cancer provided *additional* motivation to be healthier weight. Diane had recently suffered a family bereavement from breast cancer; losing weight was imperative for her as it gave her a sense that she had some control over her risk and increased her sense of well-being.

*If gaining extra weight could increase my risk of having breast cancer by 50% there was no choice. I didn’t want to live with that so there was absolutely no choice so I’m going to feel better anyway on a day to day basis and hopefully lower my risk. Well I’ve got a win-win situation (Diane, 39y, 9% weight loss with intervention).*

Not all women could easily translate information about weight and risk into action. Being informed that weight is an influential and contributory factor in breast cancer risk, did not motivate some women who instead experienced feelings of guilt and self-blame that they were not doing what they could to reduce risk. This would be a particular problem if these women were to develop breast cancer.

*She’s got 3 young children [friend diagnosed with breast cancer] and said ‘have I done this to myself because I’m fat and she said am I depriving my children of a mother because I like my food and I’m overweight?’ And I said ‘[friend’s name] don’t be stupid’, but part of me thought I could be doing that to my family and that frightens me to death (Kate, 40y, 4% weight gain with intervention).*

Despite feelings of guilt, most women in this group still valued the information that weight is a modifiable risk factor, believing that this would attenuate further weight gain or motivate a future weight loss attempt. Joan projected how she would feel if they were diagnosed with breast cancer and had not known this.

*I’d rather someone help me and tell me now than help me when I'm on the operation table having an operation. It’s the real world. It’s no good someone telling me later when it’s too late. (Joan, 44y, 3% gain during study).*

#### Responsibility for family’s health

Women reflected on how they would disseminate advice on maintaining a healthy weight to sisters and daughters who may also have an increased risk of breast cancer because of the family’s history. Nicola reported that sharing knowledge about the link between breast cancer and weight appears to have an impacted on her sister’s lifestyle behaviours.

*She’s [her sister] tried to cut down her weight a bit as well and exercise… and the alcohol, cutting down on alcohol …. I think has been particularly proven recently and I've been passing some of that on to her as well (Nicola, 41y, 7% weight loss with intervention).*

Women were very aware of the key role they have in determining their daughter’s dietary choices. Participants did not feel it is appropriate to discuss the links with diet and cancer with younger children. Hannah has two daughters less than 10 years of age and reflected on her own battle with weight and its implications for her cancer risk. She was determined to improve her family’s diet which was largely based around high fat convenience foods in order to ensure her daughters did not develop eating habits which were difficult to alter later in life.

*Well because I know that I’ve found it so hard at my age to make these changes. I think the longer you get into these bad eating habits the harder it is. So I don’t want her to have the same struggle that I’ve had when she’s older (Hannah, 42y,* weight stable with intervention*).*

Women with teenage daughters stated that they would be more likely to discuss the importance of a healthy lifestyle and avoiding excessive weight gain in preventing disease. As Nicola and Nina explain, there was evident concern to avoid making their daughters worry about weight gain and mothers were anxious to avoid precipitating an eating disorder.

*My daughter’s quite interested in health issues anyway. I think some of the message is getting across but then you don’t want to make teenage girls become too obsessive, which she doesn’t show any signs of, but you have got to be careful I think (Nicola, 46y, 7% weight loss with intervention).*

*So it’s knowing how to manage that transition, to manage to get her [teenage daughter] to eat healthily but not make a big deal about it and I spoke to [dietitian’s name] about it and she was quite helpful really in giving me some guidance on that (Nina, 39y, 15% weight loss with intervention).*

## Discussion

We have explored how women with a family history understood advice that weight loss may reduce their breast cancer risk in women following risk counselling and a 12-month weight loss intervention. Participants received intensive one to one counselling on the links between weight and risk from doctors and a research dietitian in a specialist FHC. Despite this advice their understanding of factors they consider to be influential to their cancer risk were largely informed by social networks, media reports and personal experiences of significant others diagnosed with breast cancer, indicating that for many women specialist counselling does not override these deeply held beliefs. Beliefs are an important construct in behaviour change theories. The theory of planned behaviour argues [27] that beliefs about a given behaviour are based on the knowledge that that behaviour (in this instance dietary restriction and exercise) will produce a given outcome (breast cancer risk reduction). Within this model, behavioural beliefs sit alongside social normative influences and perceived behavioural control [27] and we saw evidence for each of these three predictive factors within our interviews. A subjective appraisal of the efficacy of behaviour to produce the required outcome can determine what illness perceptions that individual holds going forward (Leventhal’s self-regulation theory) [28,29]. For women whose beliefs were constructed in the context of family members’ cancer experience and the messages they internalised through mass media communication, their illness perception of breast cancer risk was difficult to override through the dietitian’s counselling. Some women appeared to struggle to understand precisely how weight affected the development of breast cancer. The lack of a simple, coherent explanation cast doubt on the importance of weight to risk and dis-incentivised weight loss for risk management. It is uncertain whether beliefs expressed by women who did not lose weight were driving their lack of adherence to the intervention or whether they were reported to justify and explain their lack of success.

Keogh et al. suggest that some women with a family history see little value in making lifestyle changes when they remain well [21] and Rees et al. suggest this is because genetic predisposition means they remain at risk despite making lifestyle changes [30]. We have shown women who are counselled on the link between weight and cancer do gain a sense that excess body fat would further increase their cancer risk, and this is also as reported by others [31]. Women who lost weight were positive about the information given since they gained a sense of control over their risk and were doing something to manage it. Education on other breast cancer risk factors is however crucial to avoid any misconception that weight control can provide absolute control over risk [32], thus potentially decreasing anxiety in women who are unsuccessful in losing weight. Our data show that some women anticipate feelings of guilt if they were to be diagnosed with breast cancer whilst being overweight, highlighting the need for expert counselling, education and support on managing modifiable risk factors.

Excess weight in the pre and post-menopausal years is consistently linked to the development of postmenopausal breast cancer [7-9], whilst the link with premenopausal breast cancer is more complex and less established [3,4]. Premenopausal women in our FHC are counselled on the link between weight, weight gain and postmenopausal breast cancer and the potential risk of reduction with weight loss. This approach is justified since post-menopausal breast cancer accounts for two-thirds of breast cancer cases amongst high risk women (with a 16-40% lifetime risk), 40% of cases amongst *BRCA1* carriers and 50% of cases amongst *BRCA2* carriers [33]. Tackling weight problems in the premenopausal years aims to prevent weight gain which is common prior to the menopause [34] and difficult to reverse [35]. Recent data has linked weight at age 35 to risk amongst the high risk women [4] and to *BRCA* carriers [16]. Our findings suggest that knowledge of weight and breast cancer risk can provide some women with additional motivation for weight loss, but have also observed tensions between women’s lifestyle choices and cancer prevention guidelines which advocate maintaining a healthy weight [2,5].

We found that some participants, particularly those who struggled to control their own weight, had strong feelings about trying to ensure their daughters did not encounter similar problems in the future. Women attending FHCs could influence siblings and future generations, as mothers with a family history have a significant role in influencing the preventive health behaviours in their daughters [36,37]. From a family systems perspective, individuals cannot be understood in isolation or without also understanding their relational links to other family members; in this way, the family provides a platform of communication about shared family beliefs, adaptation, and coping [38]. This is important when examining beliefs about breast cancer risk that can have a potentially hereditary nature, as family rules, beliefs and interactions become habits that can impact on whether behaviour change is successful. Mothers in our study were, however concerned to avoid precipitating feelings of insecurity in their children and many were worried about eating disorders. Part of the familial risk for breast cancer may be a familial risk of obesity thus complicating the dynamic. This should be considered as part of risk counselling as our participants appreciated professional advice on how they could best support their daughters with maintaining a healthy lifestyle and weight range.

### Strengths and limitations

This study presents data from a population of women participating in a weight loss intervention aimed at reducing their risk of breast cancer. Weight gain is a sensitive issue and by utilising a one-to-one interview approach, largely conducted in participant’s homes (23 out of 24 interviews), with a female researcher of a similar age to the participants enabled us to obtain detailed, reflective accounts where women were able to explain and contextualise their beliefs about weight and breast cancer in a non-judgemental environment [39]. Our study has several limitations. Our sample mainly included women from the least deprived socio-economic backgrounds, which reflects the population in our regional family history clinic. As this paper presents an analysis of exploratory data additional studies are required to determine whether the views presented are representative of a wider context, such as women at population-risk, men, older women or those with a greater socio-economic or ethnic diversity. Further studies are required to elucidate the role of risk counselling in influencing beliefs and attitudes to weight loss and breast cancer risk, particularly in women who struggle to control their weight. Future research should also explore the extent to which behavioural change techniques, such as tailoring of information or risk counselling aid or hinder adherence to weight loss interventions.

Women with a more positive experience of the weight loss study could have been more willing to be interviewed; however, we were able to recruit similar numbers of successful and unsuccessful women and several who dropped out of the weight loss study. We feel that this range of women reflects both positive and negative experiences of weight loss intervention. Qualitative research does not aim to be statistically generalisable but gives insight into individual experiences. Our findings may be transferable to other family history populations at risk of weight-related cancers [40]. Our qualitative approach shows that counselling that weight is a risk factor can make women feel they have some control over their risk, an outcome which could not identified using validated cancer worry scores [23]. We cannot establish however whether the beliefs expressed by women who did not lose weight were driving their lack of adherence to weight loss advice or were presented to explain their lack of success.

### Implications for clinical practice

Currently, weight management is not part of clinical practice in breast cancer family history clinics, despite NICE guidelines advising health care professionals to inform women of the probable increased post-menopausal breast cancer risk from being overweight [6]. Our data highlights the need for a stronger, more consistent message. Smoking has a clear imagery link to lung cancer, but our participants show that the link between lifestyle and breast cancer is much harder to visualise. Thus there is a need for information that is supported by tailored leaflets, web based information [2,5,41] and visual aids [42] which address concerns and areas of confusion and help individuals to visualise the weight and breast cancer link. Further research is needed to clarify whether supporting women through more tailored information, for example by making clearer the additional, modifiable risk due to weight gain, could enhance women’s motivation to lose weight or avoid weight gain.

## Conclusion

Weight control is one of the few risk factors for breast cancer which is modifiable and our study has shown that advising high risk women on weight loss can give some a sense of control over their risk. It should be made clear that it is only one of the many factors associated with cancer development, avoiding any misconception that being a healthy weight could provide absolute control over cancer risk [32]. Around half of our interviewees were not able to achieve a 5% weight loss and for some information on weight and breast cancer risk may result in feelings of anxiety and guilt. The importance of avoiding further weight gain should be stressed in women whose established lifestyle habits a prevent 5% weight loss. Mothers within our study became more conscious about their daughter’s diet and exercise habits and the need to establish healthy lifestyle choices, those unable to lose weight recognised that habits were hard to change once established.

Professionals who provide genetic counselling and develop health education material should be mindful that beliefs about weight and breast cancer risk are informed by social networks, media reports and personal experiences of significant others diagnosed with breast cancer. These factors limit the effectiveness of risk counselling concerning the link between weight loss and breast cancer risk. Our study highlights the importance of providing a credible rationale for weight loss which addresses common doubts, anxieties and questions. Counselling and health education material should be tailored to facilitate understanding of both genetic and modifiable risk factors and should do more help individuals to visualise the weight and breast cancer link.

### Ethical approval

Ethical approval was obtained from the South Manchester Ethics Committee (reference 03/SM/159).
